# Tunable Mechanical Properties of Ti-Zr-Ni-Cr-V Amorphous Ribbons via Different Melt Spinning Speeds during Rapid Solidification Process

**DOI:** 10.3390/ma11060947

**Published:** 2018-06-04

**Authors:** Bing Jiang, Jianxin Wang, Lingfeng Xu, Chengyuan Qian, Tiexin Liu, Jiayu Dai, Xueling Hou

**Affiliations:** 1 Laboratory for Microstructures of Shanghai University, Shanghai 200444, China; j_gathering@163.com (B.J.); wangjianxin@i.shu.edu.cn (J.W.); xulingfeng43837@126.com (L.X.); qcy941029@shu.edu.cn (C.Q.); 2 School of Materials Science and Engineering, Shanghai University, Shanghai 200072, China; 3 Shanghai Astronomical Observatory, Chinese Academy of Sciences, Shanghai 200030, China; tiexinl@shao.ac.cn (T.L.); daijy@shao.ac.cn (J.D.)

**Keywords:** (TiZr)_0.5_(Ni_0.6_Cr_0.1_V_0.1_)_2.1_ amorphous ribbons, mechanical properties, free volume, low loading rate, serrated flow behavior

## Abstract

In this paper, the effects of different melt spinning speeds on the mechanical properties of (TiZr)_0.5_(Ni_0.6_Cr_0.1_V_0.1_)_2.1_ amorphous ribbons were studied. Tensile tests of the specimens were used to investigate mechanical behavior and mechanical properties of amorphous ribbons. The effects of cooling rate on the glass transition temperature of amorphous ribbons was discussed. The correlation between the microstructure of serrated flow behavior in stress-strain curves and melt spinning speeds of ribbons was also evaluated. In addition, when the spinning speed was 45 m/s, a large number of dense and uniform dimples appeared on the fractured surface of the specimens. Furthermore, characteristics of serrated flow behavior were obvious, which meant that Ti-Zr-Ni-Cr-V amorphous ribbons showed minor plastic behavior. It is assumed that the influence of free volume led to a serrated flow behavior of the amorphous materials, and made the amorphous material exhibit partially plastic properties. Increasing the strain rate sensitivity meant the free volume increased with the increasing spinning speed. Tensile strength (σ_b_) and elongation (δ) of samples exhibited a dramatic increasing trend with an increase in the spinning speed. In particular, Ti-Zr-Ni-Cr-V amorphous ribbons showed better mechanical properties, namely the tensile strength of the amorphous ribbon samples significantly increased from 321 MPa at a spinning speed of 30 m/s to 675 MPa at a speed of 45 m/s. The elongation increased from 0.53% at a speed of 30 m/s to 1.29% at a speed of 45 m/s.

## 1. Introduction

Metallic glass does not have defects, such as grain boundaries or dislocations in the crystal, leading to its uniformity on a very small scale and achieving high strength and partial plasticity [1]. The unique structural characteristics of metallic glass give it a completely different plastic deformation mechanism and excellent performance, including excellent corrosion resistance and soft ferromagnetic properties. Therefore, metallic glass has shown a very broad range of prospective applications in many fields, including military, aerospace, and civil applications.

Mechanical properties are crucial for amorphous alloys. Metallic glasses have high strength and low elastic modulus (E) in comparison with metallic crystals. Furthermore, the breaking strength of amorphous alloys is much higher than traditional crystalline materials [2]. Since Au_75_Si_25_ amorphous alloy was first prepared in the 1960s [3], there have been many studies on bulk amorphous alloys, such as iron-based [4], aluminum-based [5], nickel-based [6], and copper-based [7] amorphous alloys, among others. To find ways of improving the mechanical properties of metallic glasses, a large number of studies have been carried out. Amorphous ribbons possess not only excellent magnetic properties, but also excellent mechanical properties [8].

Many studies have been done to improve the plasticity of amorphous alloys, including adding elements or changing the proportion of alloy components [9,10,11]. Amorphous alloys have been widely used in surface coating due to their excellent surface properties [12]. For example, the yield strength of Zr-based amorphous films reaches 2.4 GPa [13]. The effects of adding hydrogen to Ti-Zr ribbons on mechanical properties have also been researched. Tensile strength of Ti_50_Zr_25_Co_25_ melt-spun ribbons upon electrolytic hydrogenation reaches about 1500 MPa [14]. The mechanical properties of amorphous ribbons have also been studied. Elastic modulus of Mg-based amorphous ribbons is about 50 GPa [15]. Tensile strength of Ti-Ni ribbons with the spinning speed of 51 m/s is 350 MPa and its elongation is 1.2%, and the maximum fracture strain (4.6%) was obtained from the ribbons annealed at 738 K [16].

(TiZr)_0.5_(Ni_0.6_Cr_0.1_V_0.1_)_2.1_ amorphous ribbons and their mechanical properties were investigated for the first time in the present work, in which the relationship between amorphous mechanical properties and different spinning speeds of (TiZr)_0.5_(Ni_0.6_Cr_0.1_V_0.1_)_2.1_ amorphous ribbons from 30 m/s to 45 m/s was studied. The effects of cooling rate on the glass transition temperature of amorphous ribbons was also discussed. Some systematic work has been done on the effect of free volume on mechanical properties. Tensile strength and elongation of tensile testing, strain rate sensitivity, and activation volume of nanoindentation were also studied.

## 2. Materials and Methods

The alloys with nominal composition of (TiZr)_0.5_(Ni_0.6_Cr_0.1_V_0.1_)_2.1_ were prepared by an arc-melting method under a high-purity argon atmosphere. Each sample was re-melted four times to ensure homogeneous compositions of ingots. Ribbons were prepared using a melt spinner equipped with a Cu wheel rotating at a speed of 15~45 m/s.

The phases in the ribbons were identified by X-ray diffraction (XRD) using CuKα radiation, model D/max-2200. The ribbons were pulverized using an agate mortar before testing with model D/max-2200. In addition, the scanning speed was 2°/min and the range was 20–90°. Thermal properties of each amorphous ribbon were analyzed by differential scanning calorimeter (DSC, PE Diamond) at heating speed of 20 K/min, in the argon atmosphere. The microstructural analysis was carried out by transmission electron microscopy (TEM, JEM-2010 F) and scanning electron microscope (SEM) equipped with an energy dispersive spectrometer (EDS) analysis system (JSM-6700 F). There was a direct observation using an SEM of an in-situ fractured surface of amorphous ribbons subjected to the tensile test. For TEM, the sample was thinned using a double-jet electrolytic thinning device (MTP-1A) with 20% perchloric acid and 80% acetic acid, and sample diameter was 2 mm. Tensile strength of each alloy ribbon was measured by a universal testing machine operating at a loading rate of 8.3 × 10^−6^ m/s, model Zwick Biaxial-10 kN, in the air atmosphere. Each amorphous ribbon with uniform width and thickness was selected to produce a sample with a standard size; the width and length of the standard size was 4 mm and 55 mm, respectively. In addition, the thickness of ribbons with the spinning speed of 30 m/s, 35 m/s, 40 m/s, and 45 m/s was 0.3 mm, 0.16 mm, 0.09 mm, and 0.04 mm, respectively. The nanoindentation was tested at room temperature, and Berkovich tip mounted on an MTS Nano Indenter XP head. The loading rate was 0.05 mN/s, 0.25 mN/s, 0.5 mN/s, and 1 mN/s, and the loading force was 5 mN.

## 3. Results and Discussion

**Figure 1:**
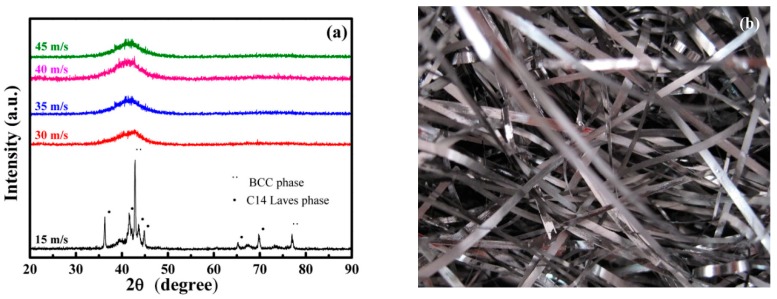

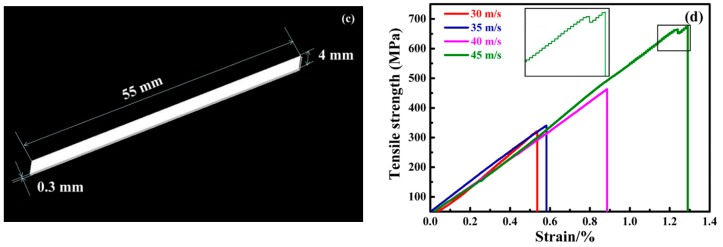
(**a**) XRD patterns of (TiZr)_0.5_(Ni_0.6_Cr_0.1_V_0.1_)_2.1_ amorphous ribbons with different spinning speeds; (**b**) shapes of (TiZr)_0.5_(Ni_0.6_Cr_0.1_V_0.1_)_2.1_ amorphous ribbons; (**c**) the model of ribbons in the tensile test; (**d**) stress–strain curves of amorphous (TiZr)_0.5_(Ni_0.6_Cr_0.1_V_0.1_)_2.1_ alloy with different spinning speeds.

Figure 1a presents the XRD patterns of (TiZr)_0.5_(Ni_0.6_Cr_0.1_V_0.1_)_2.1_ amorphous ribbons with different spinning speeds, confirming the phases and crystal structures of the investigated samples. This figure shows that crystalline phases of the 15 m/s spinning speed sample were determined to be BCC and C14 Laves phases. The other Ti-Zr-Ni-Cr-V ribbons showed a disordered structure in terms of atomic arrangement, and a main hole without a sharp diffraction peak indicated the amorphous structure of the ribbons. From XRD patterns of amorphous ribbons, we can see that 2θ shifts toward low angle with the increasing spinning speed. The maximum peak intensity of the amorphous scattering peak corresponds to the coherence distance of the quasi-periodic particles in the amorphous phase. The maximum peak intensity is usually used to compare the degree of dispersion between particles. Therefore, we assumed that the larger 2θ is, the less free volume there is [17]. In this case, the free volume of ribbons with the spinning speed of 45 m/s is significantly higher than those with a 30 m/s spinning speed.

Figure 1b shows the shapes of (TiZr)_0.5_(Ni_0.6_Cr_0.1_V_0.1_)_2.1_ amorphous ribbons with different spinning speeds. Figure 1c shows the model of ribbons with the spinning speed of 30 m/s in the tensile test. In particular, the length, width, and thickness of amorphous ribbons were 55 mm, 4 mm, and 0.3 mm, respectively. In addition, the thicknesses of ribbons with the spinning speed of 30 m/s, 35 m/s, 40 m/s, and 45 m/s were 0.3 mm, 0.16 mm, 0.09 mm, and 0.04 mm, respectively.

Figure 1d presents the tensile stress-strain curves of (TiZr)_0.5_(Ni_0.6_Cr_0.1_V_0.1_)_2.1_ amorphous ribbons at room temperature under different spinning speeds from 30 m/s to 45 m/s. Tensile strength was found using the breaking strength of materials at the room temperature. The elongation was measured at the maximum strength point. When the spinning speed was 45 m/s, amorphous ribbons showed a serrated flow behavior, in which case the amorphous ribbons showed good toughness. Table 1 shows that tensile strength and elongation of amorphous ribbons increased, while the elastic modulus decreased, as the spinning speed increased. When the spinning speed was 30 m/s, tensile strength, elongation, and elastic modulus were 321 MPa, 0.53%, and 67.9 GPa, respectively. When the spinning speed increased to 45 m/s, the ribbons’ tensile strength and elongation reached 675 MPa and 1.3%, respectively, while the elastic modulus dropped to 55.4 GPa. The average tensile strength of amorphous ribbons with the spinning speed of 30 m/s, 35 m/s, 40 m/s, and 45 m/s reached 321 ± 37, 340 ± 33 MPa, 457 ± 37 MPa, and 675 ± 32 MPa, respectively. The average strains of amorphous ribbons with the spinning speed of 30 m/s, 35 m/s, 40 m/s, and 45 m/s were 0.53% ± 0.04%, 0.58% ± 0.05%, 0.89% ± 0.05%, and 1.29% ± 0.08%, respectively.

When the thickness of ribbons is much smaller than both the length and the width, size effects occur, which will affect the mechanical behavior of amorphous ribbons [13,18]. Therefore, with decreasing thickness of amorphous ribbons, size effects may also lead to an increase in the tensile strength and elongation. The increase of the speed of ribbons will lead to a reduction in the thickness of samples obtained. This is one of the reasons why the tensile strength and elongation of the ribbons with the spinning speed of 45 m/s are higher than those with a spinning speed of 30 m/s, 35 m/s, and 40 m/s because the former is thinner than the latter three. However, even the thickness of ribbons with the speed of 45 m/s was much greater than the nanoscale critical dimensions [19], so the deformation mode was dominated by the shear bands and did not transition to uniform plastic deformation.

Low elastic modulus indicates enhanced toughness. Elastic modulus reflects the magnitude of the binding force between atoms. Due to the random arrangement of atoms, the atomic spacing was smaller compared to the crystalline metal, and therefore the binding force was smaller. Therefore, less stress could result in a larger displacement in the amorphous ribbons, causing a larger elastic deformation and smaller elastic modulus. The increased spinning speed increased the free volume and reduced the viscosity [20]. The annihilation volume caused by the structure relaxation could not completely offset the free volume under stress [21,22]. The free volume had increased in certain areas.

**Figure 2:**
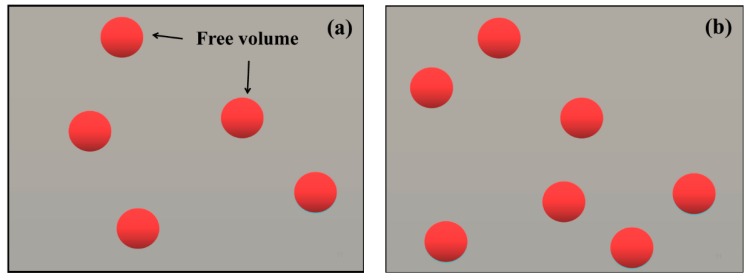

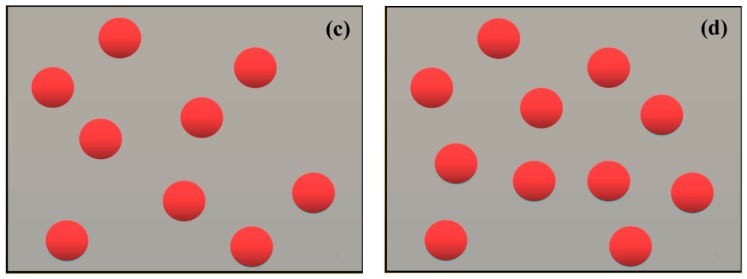
Schematic diagrams of the free volume for (TiZr)_0.5_(Ni_0.6_Cr_0.1_V_0.1_)_2.1_ ribbons with different spinning speeds. The spinning speed were (**a**) 30m/s, (**b**) 35 m/s, (**c**) 40 m/s, and (**d**) 45 m/s, respectively.

Figure 2 shows the schematic diagrams of the free volume for the ribbons with different spinning speeds. We assumed that the free volume of the sample increased as the spinning speed increased, which led to an increase in tensile strength and elongation, and to a decrease in the elastic modulus [23,24,25]. For tensile stress-strain curves of the samples, the increase of the free volume may result in the generation of serrated flow behavior. Therefore, we postulate that when the spinning speed was 45 m/s, the free volume may have been the reason for the specimen displaying plastic behavior.

**Figure 3:**
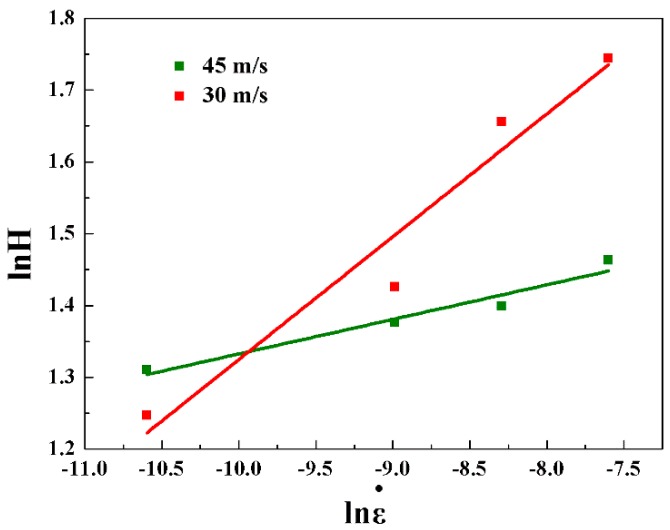
Nanoindentation results of the hardness as function of the strain rate for (TiZr)_0.5_(Ni_0.6_Cr_0.1_V_0.1_)_2.1_ amorphous ribbons with spinning speeds of 30 m/s and 45 m/s.

Figure 3 shows the nanoindentation results of the hardness as a function of the strain rate for amorphous ribbons for different spinning speeds. When the spinning speed was 45 m/s, the average strains of amorphous ribbons at the loading rates of 0.05 mN/s, 0.25 mN/s, 0.5 mN/s, and 1 mN/s were 3.72 ± 0.02 GPa, 3.96 ± 0.02 GPa, 4.05 ± 0.01 GPa, and 4.32 ± 0.01 GPa, respectively. When the spinning speed was 30 m/s, the average strains of amorphous ribbons at the loading rates of 0.05 mN/s, 0.25 mN/s, 0.5 mN/s, and 1 mN/s were 3.48 ± 0.03 GPa, 4.16 ± 0.02 GPa, 5.24 ± 0.02 GPa, and 5.72 ± 0.02 GPa, respectively.

The relationship between the hardness (H) and the strain rate ($\overset{•}{\varepsilon }$) of each amorphous ribbon was linearly fitted. The strain rate can be estimated as equal to half the loading rate [26]. The strain rate sensitivity (m) is equal to ∂lnH/∂ln$\overset{•}{\varepsilon }$. From Figure 3, we can see that the strain rate sensitivity decreased with increasing spinning speed as m decreased from 0.171 for a spinning speed of 30 m/s to 0.048 for a spinning speed of 45 m/s. In this way, a smaller strain rate sensitivity means more free volume with the increasing spinning speed. The activation volume (ΔV) is approximately to 3√3kT/mH, where k is the Boltzmann constant and T is the temperature [6]. Therefore, the activation volume is around 0.027 nm^3^ for a spinning speed of 30 m/s and around 0.111 nm^3^ for a spinning speed of 45 m/s. These results show that the free volume increases with the increasing spinning speed.

**Figure 4:**
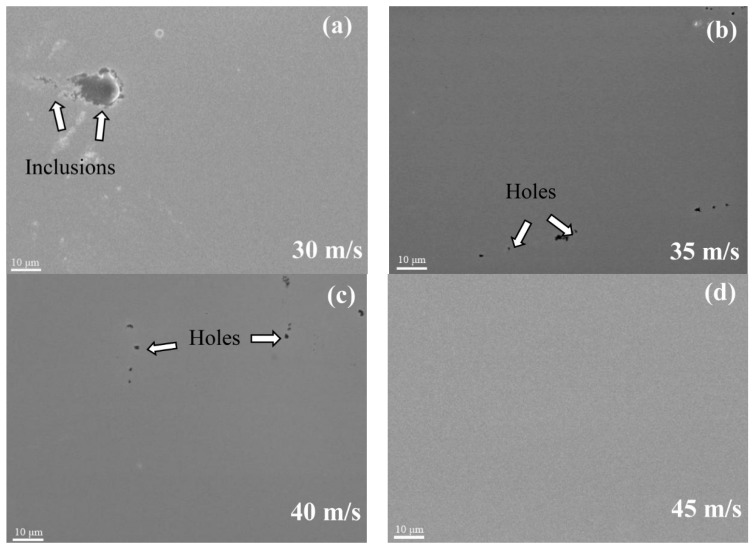
SEM images of the amorphous (TiZr)_0.5_(Ni_0.6_Cr_0.1_V_0.1_)_2.1_ ribbons’ surfaces with different spinning speeds. The spinning speed were (**a**) 30 m/s, (**b**) 35 m/s, (**c**) 40 m/s and (**d**) 45 m/s, respectively.

Figure 4 shows the SEM pictures of the amorphous (TiZr)_0.5_(Ni_0.6_Cr_0.1_V_0.1_)_2.1_ ribbons’ surfaces with different spinning speeds. Figure 4a,b shows that when the spinning speeds are 30 m/s and 35 m/s, inclusions, scratches, and holes are present on the surface of the ribbons. In this case, surface roughness is high. The surface quality became better when the spinning speed was 40 m/s (Figure 2c). However, there were still some local pores and the composition was not uniform. When the spinning speed increased to 45 m/s (Figure 4d), defects, such as inclusions and voids, were not found, which implied that the composites were successfully fabricated by the rapid solidification process. As a result, surface quality was greatly improved and the composition was more uniform. From this, we conclude that the surface quality and uniformity of the (TiZr)_0.5_(Ni_0.6_Cr_0.1_V_0.1_)_2.1_ amorphous ribbons increase with increasing spinning speeds. When the spinning speed was high, the amorphous solid formed by rapid solidification of the alloy was more uniform. Surface quality, uniformity, and void size of amorphous ribbons affected the strength of the ribbons. When surface quality was poor, tensile strength of amorphous ribbons was low, and vice versa. Consequently, with the increase of spinning speed, the mechanical properties of amorphous (TiZr)_0.5_(Ni_0.6_Cr_0.1_V_0.1_)_2.1_ ribbons increased.

**Figure 5:**
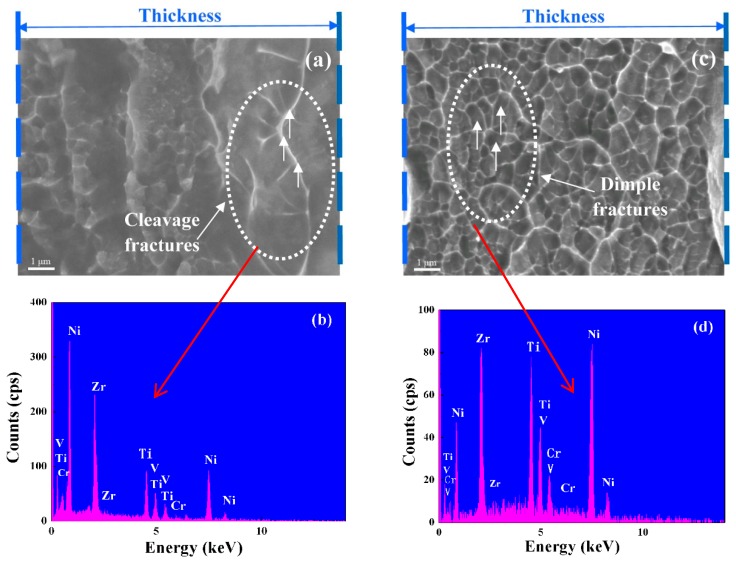
Fracture SEM images and EDS analysis of (TiZr)_0.5_(Ni_0.6_Cr_0.1_V_0.1_)_2.1_ ribbons with spinning speeds of 30 m/s and 45 m/s; (**a**) 30 m/s microfracture; (**b**) 30 m/s EDS; (**c**) 45 m/s microfracture; (**d**) 45 m/s EDS.

Figure 5 shows the SEM pictures of the tensile fracture of (TiZr)_0.5_(Ni_0.6_Cr_0.1_V_0.1_)_2.1_ amorphous ribbons with the spinning speeds of 30 m/s and 45 m/s, and the results of corresponding EDS analysis. SEM pictures showed that the fracture morphology was dominated by cleavage fractures and vein-like patterns. Figure 5a shows that cleavage fractures appeared near the free surface of the ribbons and vein-like patterns appeared near the other side. The reason is that the cooling rate of the free surface is different from the roller surface. The low cooling rate of the free surface led to low amorphous content, while the cooling rate of the roller surface was high, which led to high amorphous content. The difference in amorphous content led to the microstructure change of the ribbons and different fracture morphology. At the same time, obvious protruded riblets appeared on the edge of vein-like patterns (Figure 5c). According to the non-uniform deformation mechanism of amorphous alloys [27], the amorphous alloys at room temperature produced a shear slip band under the influence of stress. Figure 5a,c shows that the dimples with the spinning speed of 45 m/s are smaller and denser than those at the spinning speed of 30 m/s. By EDS analysis of the fractures (Figure 5b), it was found that when the spinning speed was 30 m/s, an enrichment of Zr and a depletion of Cr occurred. Furthermore, when the spinning speed was 45 m/s, atomic percentage of each element was basically the same as the original ratio (Figure 5d). The uniformity of the composition was also a factor that affected the mechanical properties of amorphous ribbons.

**Figure 6:**
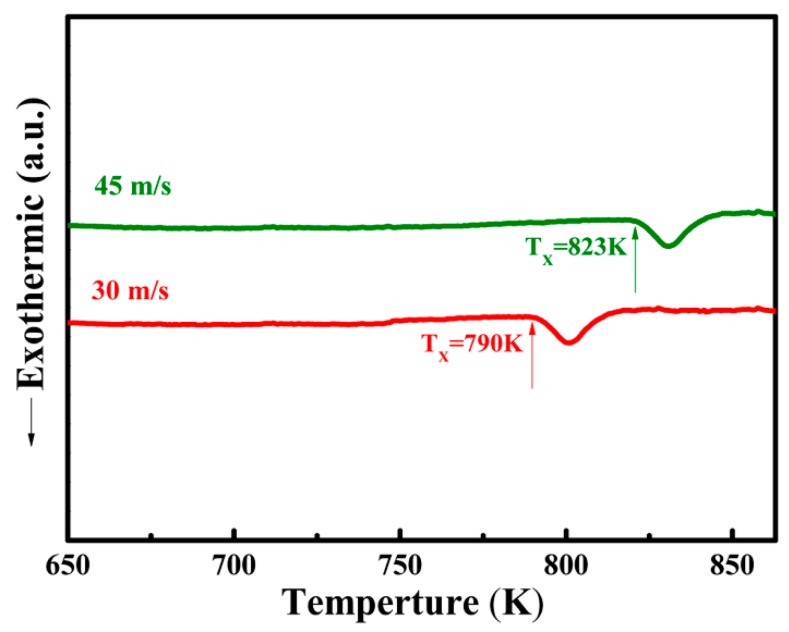
DSC curves of (TiZr)_0.5_(Ni_0.6_Cr_0.1_V_0.1_)_2.1_ alloy with spinning speeds of 30 m/s and 45 m/s.

Figure 6 shows the DSC curves for the (TiZr)_0.5_(Ni_0.6_Cr_0.1_V_0.1_)_2.1_ ribbons with different spinning speeds of 30 m/s and 45 m/s. The heating rate used in this test was 20 K/min. It can be seen from the figure that the ribbons obtained at the spinning speeds of 30 m/s and 45 m/s had an exothermic peak. Thus, the result indicated that ribbons obtained at 30 m/s and above all contained an amorphous phase, which was consistent with the XRD results in Figure 1a. It can also be seen from the figure that the glass transition temperature of the ribbons obtained at a spinning speed of 45 m/s was higher than that obtained at a spinning speed of 30 m/s, which are 823 K and 790 K, respectively. This showed that with higher spinning speed, the thermal stability of amorphous ribbons was enhanced. When the spinning speed was 45 m/s, the thermally stability contributed to the invisibility of defects on the sample’s surface. The cooling rate (R_c_) is equal to (T_m_ − T_g_)/t, where t is the cooling time required for the amorphous ribbons to go from melting point (T_m_) to glass transition temperature (T_g_). Furthermore, R_c_ is proportional to exp(V_r_), where V_r_ is the spinning speed of the ribbons [28]. Therefore, R_c_ increases with increasing V_r_, which is in accordance with Figure 6, where a spinning speed of 30 m/s produces a glass transition temperature of 790 K and a spinning speed of 45 m/s produces a glass transition temperature of 823 K. The glass transition temperature is the temperature at which the free volume reaches a certain critical value, and a faster spinning speed means more free volume. In this case, we can say that T_g_ increased with increasing V_r_. In conclusion, the glass transition temperature of the ribbons increased in agreement with the increased cooling rate of the ribbons.

**Figure 7:**
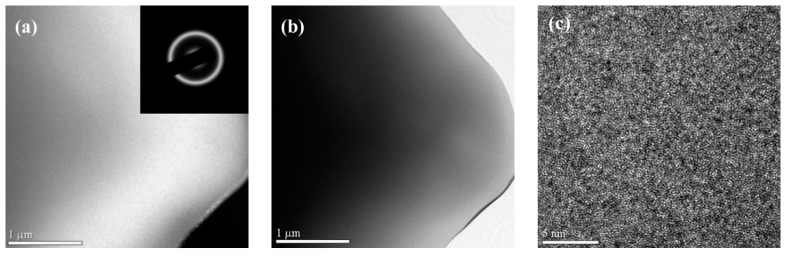
(**a**) Dark-field image of (TiZr)_0.5_(Ni_0.6_Cr_0.1_V_0.1_)_2.1_ ribbon with a speed of 45 m/s and its corresponding SAED pattern in inset; (**b**) bright-field TEM; and (**c**) high resolution TEM images of (TiZr)_0.5_(Ni_0.6_Cr_0.1_V_0.1_)_2.1_ ribbons.

Figure 7 shows TEM pictures of the (TiZr)_0.5_(Ni_0.6_Cr_0.1_V_0.1_)_2.1_ amorphous ribbons obtained at the spinning speed of 45 m/s. Figure 7c shows that the surface of the amorphous ribbon is even and flat, and no change in brightness contrast is found. No regularly arranged crystal structures in microstructure were observed, indicating a dense and uniform glassy state. The corresponding electron diffraction pattern of the selected area, shown in the inset of Figure 7a, was a halo, which is a typical amorphous electron diffraction pattern. This consistency with the XRD patterns provide further confirmation that the ribbons obtained at the spinning speed of 45 m/s were amorphous.

## 4. Conclusions

(TiZr)_0.5_(Ni_0.6_Cr_0.1_V_0.1_)_2.1_ amorphous ribbons obtained at the spinning speed of 45 m/s exhibited better mechanical properties than those obtained at a spinning speed of 30 m/s, and a certain degree of plastic deformation was observed. Tensile strength, elongation, and elastic modulus were determined to be 675 MPa, 1.3%, and 55.4 GPa, respectively. In the stretched state, (TiZr)_0.5_(Ni_0.6_Cr_0.1_V_0.1_)_2.1_ amorphous ribbons at the speed of 45 m/s showed partial plastic deformation behavior, possibly due to the creation of free volume; a faster spinning speed leads to more free volume. Furthermore, an increase in cooling rate leads to an increase in the glass transition temperature. At the same time, the high melt spinning speed not only eliminated the depletion and enrichment of the components, leading to more uniform composition of (TiZr)_0.5_(Ni_0.6_Cr_0.1_V_0.1_)_2.1_ amorphous ribbons, but also provided the ribbons with high thermal stability. To a certain extent, mechanical properties of (TiZr)_0.5_(Ni_0.6_Cr_0.1_V_0.1_)_2.1_ amorphous ribbons improved with increasing spinning speed.

## Figures and Tables

**Table 1:** Tensile strength, elongation, and elastic modulus of amorphous (TiZr)_0.5_(Ni_0.6_Cr_0.1_V_0.1_)_2.1_ ribbons at different spinning speeds.

Speed (m/s)	30	35	40	45
Tensile strength (MPa)	321	340	457	675
Elongation (%)	0.53	0.58	0.89	1.29
Elastic modulus (GPa)	67.9	64.7	59.8	55.4
